# Neurectomies for treatment of stroke-related upper extremity spasticity

**DOI:** 10.3171/2022.9.FOCVID22106

**Published:** 2023-01-01

**Authors:** Mark A. Mahan

**Affiliations:** Department of Neurosurgery, Clinical Neurosciences Center, University of Utah, Salt Lake City, Utah

**Keywords:** spasticity, partial neurectomy, tone, video

## Abstract

Limb spasticity due to central nervous system lesions can lead to substantial functional impairment. This is particularly challenging when the patient retains control of limb movements. This intraoperative video demonstrates selective neurectomies to improve spasticity. The case of a patient with left spastic hemiplegia after stroke who requested peripheral neurectomies for durable treatment of left elbow flexion spasticity is shown. The patient had substantial improvement in resting tone and achieving a relaxed, near-full extension at rest. He retains elbow flexion, which has improved in subsequent clinical follow-up evaluation. Highly selective partial denervation surgery can successfully reduce spasticity.

The video can be found here: https://stream.cadmore.media/r10.3171/2022.9.FOCVID22106

## Transcript

We are delighted to present an example of the surgical treatment for upper-extremity elbow flexion spasticity with the use of highly selective partial neurectomies.^1–5^

### 0:29 Clinical Presentation.

In this video, we provide the case of a 43-year-old man who had left-sided spasticity secondary to a right stroke in 2015 when he did not take his coumadin as directed after an aortic valve replacement surgery.

He experienced left-sided spastic hemiplegia, wherein his left upper-extremity spasticity was more severe and debilitating than his left lower extremity. As an example, he was able to walk unassisted and does not need to wear an ankle-foot orthosis. Spasticity due to stroke is frequently a complex segmental reflex. It typically involves multiple joints of the upper extremity similar to decorticate posturing.

He had been managed for 5 years with botulinum toxin injections and physical therapy, which had no long-term benefit for his spasticity.

For most patients with spasticity due to stroke, traumatic brain injury, or spinal cord injury, nonsurgical management for 6–12 months is appropriate to help identify compensatory mechanisms as well as recovery from the primary injury. However, prolonged treatment with botulinum toxin therapy is associated with progressive joint contracture, which leads to more profound deformities and loss of function. If there is a question of whether the joint position is related to tendon or bone contracture, or due to neuromuscular tone, highly selective motor nerve blockade with the use of local anesthetics delivered through ultrasound-guided injections can be helpful to assess the degree that highly selective partial neurectomies may be beneficial.

### 1:55 Preoperative Status.

The images here demonstrate typical elbow flexion, pronation, wrist and finger flexion spasticity; however, because the spasticity arises from a segmental reflex, which involves multiple spinal levels simultaneously, highly selective partial neurectomies performed for proximal muscles can lead to changes in the tone of distal musculature. This is caused when the neurectomy reduces afferent input from the sensory components of the muscle, such as muscle spindles and muscle spray endings. This leads to altered inputs in the interneuron circuits of the spinal cord, which then changes the output of the different fibers. Thus, the effect of proximal neurectomies on distal musculature can be difficult to predict. Therefore, it is my preference to perform highly selective partial neurectomy surgeries independently and not perform procedures in the same setting. Therefore, we will only perform elbow flexion surgery in this video.

### 2:50 Intraoperative Identification of Nerve Branches.

Appropriate identification of all of the nerve branches to the spastic muscle and/or movement is critical. In the still image, care has been taken to identify the typical branches to the humeral elbow flexors, including the typical configuration of the two nerve branches to the biceps—one proximal, one distal—as well as a single nerve branch to the brachialis. The nerve to the brachialis requires internal neurolysis from the lateral antebrachial cutaneous nerve. Importantly, the proximal musculocutaneous nerve is also clearly identified, as it is necessary to stimulate it as proximal as possible to confirm that all distal branches have been successfully denervated at the conclusion of the case.

### 3:28 Procedure.

The steps in this procedure are identification of the nerve branch to brachialis, specifically the separation from the lateral antebrachial cutaneous nerve. As shown in this still from the operative video, the nerve to the brachialis is slightly more lateral and inferior to the lateral antebrachial cutaneous nerve. The nerve to the brachialis is selectively isolated in the blue vessel loop as seen in the video still. The next step in the procedure is then isolation and elevation of the nerve to the brachialis with epineurial stitches using 4-0 Neurolon suture. Two fascicles within the nerve to brachialis are identified. Once elevated, the epineurium is then opened with microscissors to facilitate exposure of the fascicles within the nerve. Electrical stimulation at low current settings is then used to the assess the spastic response of the muscles in response to stimulation. The perineurium of the fascicles is then opened with an 11 blade, and the endoneurial contents are seen pooching out, with careful observation of the spiral bands of Fontana. Once the nerves have been isolated, the 11 blade is then used to dissect within the endoneurial contents to perform a highly selective partial neurectomy. Care is taken to only grasp the portion of the nerve that are slated for removal with the pickups. In this situation of severe spasticity, approximately 80% of the cross-sectional area of the nerve will be sacrificed in order to reduce the afferent information that leads to spastic contraction of the brachialis.

Once one fascicle is treated to approximately 80% of its cross-sectional area, attention will then be turned to the other fascicle, which also showed spastic hyperactivity. Again, the same maneuvers are performed with careful opening of the perineurium and then delineation of the endoneurial contents and partial sectioning of the endoneurial contents to approximately 80% of their cross-sectional area. As well, care is taken to not crush the fascicles that are destined to be left intact for preservation of motor control to the brachialis at the end of the surgery. Once the sectioning is determined to be complete, stimulation is then performed to assess response, with stimulation distally providing profound contraction of the muscle and stimulation proximally demonstrating no meaningful contraction of the brachialis despite continuity of the nerve to the muscle. As noted here, approximately a 1-cm section of the nerve is disrupted to minimize the amount of regeneration across the sectioned portions of the nerve. In general, our experience has been that patients do retain motor control despite the loss of conductivity at this time and tend to go on to have meaningful reduction of their spasticity at long-term follow-up.

### 6:25 Three-Month Postoperative Evaluation.

Postoperative evaluation at 3 months demonstrated substantial improvement in resting tone, with the ability to have the arm in a relaxed, near-full extension when at rest. The patient retains elbow flexion, with approximately MRC grade 4 strength, which has improved in subsequent clinical follow-up evaluation. The patient subsequently requested to undergo wrist pronation and finger flexion denervation procedures, because of the successful reduction of spasticity from the musculocutaneous highly selective partial denervation surgery.

I recommend starting limb stretching exercises as soon as tolerated by the patient, immediately after surgery. Physical therapy can be an important adjunct, particularly in facilitating stretching exercises as well as focus on reestablishing movements that were previously difficult due to the spasticity.

The primary challenge of highly selective partial neurectomy surgery is achieving an appropriate balance of denervation without excessive weakening of the motor pathways to the muscle. When at least 20% of the motor pathway is left intact, motor recovery is nearly entirely assured, due to the phenomenon of collateral sprouting of motor units. Fortunately, because this surgery interrupts the pain transmission from the muscle or because of neglect or loss of sensation from the primary CNS pathology, it is exceptionally rare for patients to describe new postoperative pain. Rather, the typical result is reduction in pain due to the reduction in muscle spasm.

Thank you very much for watching our video.

## Acknowledgments

We thank Vance Mortimer for assistance in preparing the video and Kristin Kraus for editorial assistance.

## Disclosures

Dr. Mahan: personal fees from joimax, Richard Wolf Spine, and SPR Therapeutics, outside the submitted work; and grants from NIH and NREF, outside the submitted work.
